# The Growth and Scope of Voluntary Hospitals in London

**Published:** 1897-10-02

**Authors:** 


### The Institutional Workshop.

### THE GROWTH AND SCOPE OF VOLUNTARY HOSPITALS IN LONDON.

(From a Correspondent.)

### XI.—HOSPITAL STATISTICS IN 1809.

The figures are very scanty which deal with this period of hospital work, and they are often rendered of less use by the custom which then prevailed of stating the numbers benefited by the institution since its foundation rather than those who had been under treatment during the past year. The number of occupied beds can only be judged from the number of patients remaining under treatment on a given day, which may easily, of course, be misleading if adopted as an average. Concerning Guy's Hospital no figures are given, and it appears from Thomas Pennant, who wrote a history of London at the end of the last century, and repeatedly applied at Guy's for information without result, that this institution had at this period a reputation for unwise and unmannerly secretiveness.

The following table gives the number of patients in and out, and those under cure in the year 1808-9, with the apparent average length of stay in hospital, calculated on the basis of those in the hospitals mentioned on a given day:—

| | In-patients. | Under cure. | Average length of stay. | Out-patients. |
|---|---|---|---|---|
| St. Bartholomew's | 3,849 | 440 | 41·7 days | 4,540 |
| St. Thomas's | 2,789 | 417 | 54·5 days | 4,322 |
| Westminster | 627 | 73 | 42 4 days | 687 |
| St. George's | 1,450 | 146 | 36·7 days | 1,121 |
| London | 1,406 | — | — | 877 |
| Middlesex | 555 | — | about 2 months | 522 |

The figures for 1895 are as follows:—

| | In-patients. | Under cure. | Average length of stay. | Out-patients. |
|---|---|---|---|---|
| St. Bartholomew's | 6,774 | 548 | 30·4 days | 159,063 |
| St. Thomas's | 6,150 | 366 | 21·4 days | 112,056 |
| Westminster (1894) | 2,934 | 181 | 22·8 days | 24,247 |
| St. George's | 4,191 | 280 | 24·3 days | 28,392 |
| London | 10,559 | — | — | 152,411 |
| Middlesex | 3,404 | — | 28·0 days | 41,706 |

The length of stay admitted of wide variations, although it can hardly have outdone the record of a Colonial general hospital, which recently filled in the usual form, "Longest stay of any patient during the year—365 days." That chronic cases were allowed to linger on for months is clear from the direction that patients who had been over two months in the ward should procure a second letter of recommendation. That they were occasionally admitted for very slight disorders, and kept but a short time, may be inferred from a recommendation made in 1816, that "in the future statements of patients all cases of tooth-drawing and bleeding be omitted in the list of accidents."

It will be seen on comparing the above two tables that the growth of the out-patients' departments has been out of all proportion greater than that of the in-patients. At St. Bartholomew's and St. Thomas's the present number of in-patients barely exceeds that in 1809 if the proportionate length of stay be allowed full weight. At St. Bartholomew's the number of out-patients is 35 times as great, and at St. Thomas's about 26 times. Westminster has about doubled her in-patients in proportion to their stay, but her out-patients have been multiplied by 35. St. George's, again, has about doubled the in-patients, but the out-patients are 25 times as many. The London, which has about three times the number of beds, receives 173 times the number of out-patients, and Middlesex about 80 times.

Turning to the dispensaries, the increase in the number of patients is seen to be in an entirely different ratio to that of the out-patients' departments in hospitals. The following table gives the number attending and attended in 1808-9 and 1895 respectively, the number having been obtained by striking an average from the total relieved since the foundation. Statistics are only available in a few instances. Taking all the dispensaries together at the end of the century, Dr. Lettsom estimates they were sufficient for the relief of 50,000 annually.

Number of Out-patients and those Attended in their Own Homes.

| | 1808 9. | 1895. |
|---|---|---|
| Royal Eye Infirmary | 2,523 (67 in-patients) | 26,290 (2,184 in-patients) |
| Royal Gen. Dispensary | 3,634 (average for 38 years) | 4,083 (+2,623 home visits) |
| London Dispensary... | 2,997 (average for 33 years) | 2,600 |
| Public Dispensary... | 2,131 (average for 25 years) | 3,378 (+471 visited at home) |
| Surrey Dispensary... | 2,892 (average for 32 years) | 12,377 |
| Western Dispensary | 1,438 (average for 19 years) | 8,774 (inc. Provdnt. Branch) |
| Westmnstr. Gen. Dis. | 3,258 (average for 34 years) | 6,505 |

The largest increase among the dispensaries is the Western, which has multiplied its patients by six. The others show merely a normal rate of progress, and in one case (the London Dispensary) there is actually a falling off. The Royal Eye Infirmary, the only special hospital then existing besides of those for lying-in and for infectious disorders, reverses the condition of affairs at the general hospitals, and shows an increase of only ten times the number of out-patients, and of in-patients no less than 32 times the number. We shall hope to trace later on the causes which have led to the disproportionate growth of the out-patient departments in general hospitals. Meantime, a few words may be said on the question of the comparative cost of institutions for the sick, then and now.

The figures relating to the cost per bed and per patient are fragmentary, and, owing to the fact that ordinary expenditure is not distinguished from extraordinary, may easily be misleading. It has not been possible to make any deductions for the cost of out-patients.

| Average cost. | 1809. | | 1895. | | |
|---|---|---|---|---|---|
| | Per bed. | Per patient. | Per bed. (Less out-patients.) | Per bed. On total ordinary expenditure. | Per patient. |
| St. Bart.'s ... | £28-9 | £3 | £98 | £122 | £8 |
| St. Thomas's... | £19 | £3 | 101 | £122 | £6½ |
| Westminster... | £44 | £5 | 67 | £78 | £4½ |
| St. George's... | £40 | £4 | 92 | £99 | £6½ |

It will be seen that the cost per patient has not increased in anything like the same ratio as the cost per bed, and this is easily understood by reference to the table on opposite page giving the average length of each patient's stay in the hospital.

So many causes have combined to produce the higher rate of the present day, and so little is known of the domestic arrangements in institutions in the past that it would be unsafe to attempt even to account for the wide disparity in the cost as above. It may, however, be safely asserted that in whatever direction the hospitals of old succeeded in curtailing their expenditure, it was not done by rigid attention to details and efficient organisation. These arts, applied to institution life, are essentially of modern growth. Whenever the curtain is lifted, and a glimpse obtained of the old-fashioned ways, it reveals almost certainly a vision of waste and confusion in the household which the puzzled managers, in the dearth of any properly qualified officials, could meet only by futile regulations. Such a picture is revealed in an old inquiry into increased expenditure by the governors of Middlesex Hospital, when it appeared that in the year 1816 no fewer than 36,100 leeches had been used in the hospital, and the consumption of 1,000 bottles of wine during the same period seems a mere trifling item in comparison.

No clue is given to the cost per patient of the dispensaries. The following, however, is Dr. Lettsom's estimate of the expense of keeping up these establishments, and it may be believed he wrote from actual experience. The period was towards the close of last century :—

| | |
|---|---|
| Three physicians and three surgeons ... ... | £600 |
| Apothecary... ... ... ... ... ... | 80 |
| Two assistant apothecaries ... ... ... | 80 |
| Secretary ... ... ... ... .. ... | 40 |
| Collector ... ... ... ... ... ... | 100 |
| Drugs, &c. ... ... ... ... ... ... | 500 |
| House rent ... ... ... ... ... ... | 120 |
| Miscellaneous ... ... ... ... ... | 100 |
| | £1,620 |

The contrasts in the profession were then as acute as now, if his estimate of the salary of his six physicians and surgeons be taken as a correct average, when it is borne in mind that his own "professional emoluments as first physician in London" averaged from £5,000 to £12,000 a year.

